# Correction: Consistent global structures of complex RNA states through multidimensional chemical mapping

**DOI:** 10.7554/eLife.10095

**Published:** 2015-07-21

**Authors:** Clarence Yu Cheng, Fang-Chieh Chou, Wipapat Kladwang, Siqi Tian, Pablo Cordero, Rhiju Das

Cheng CY, Chou FC, Kladwang W, Tian S, Cordero P, Das R. 2015. Consistent global structures of complex RNA states through multidimensional chemical mapping. *eLife* **4**:e07600. doi: 10.7554/eLife.07600

Published 2 June 2015

Table 1 has been corrected. The original manuscript used a previously published formula from Hajdin et al. *RNA* (2010) for estimating the p-value of a 3D RNA structure prediction but calculated these p-values using the incorrect mean RMSD values. When the correct parameters are used, the p-values of our predictions decrease, i.e. the estimated significances are increased. The conclusions of the manuscript are unchanged.

The corrected table is shown here, with changes highlighted:

Table 1 Corrected. Benchmark of multidimensional chemical mapping on RNAs with crystal structures

**Table:** 

RNA	Length	M^2^/Rosetta (no MOHCA, control)		MCM	
		RMSD to crystal (Å) (accuracy)	p-value§	RMSD to crystal (Å) (accuracy)	p-value§
*Tetrahymena* ribozyme P4–P6 domain	158	38.3	>0.9	8.6	<1.0 × 10^−16^
V. cholerae cyclic-di-GMP riboswitch aptamer, ligand-bound*	89	11.3	2.6 × 10^−3^	7.6	6.3 × 10^−7^
F. nucleatum double glycine riboswitch ligand-binding domain, ligand-bound*	159	30.5	>0.9	7.9	<1.0 × 10^−16^
S. thermophilum adenosylcobalamin riboswitch aptamer, ligand-bound*	168	17.1†	5.3 × 10^−7^	11.9	4.0 × 10^−15^
Class I ligase	127	26.3	>0.9	14.5	6.8 × 10^−5^
Class I ligase, core domain‡	87	14.0	0.13	11.1	3.1 × 10^−3^
D. iridis lariat-capping ribozyme	188	9.6†	<1.0 × 10^−16^	8.2	<1.0 × 10^−16^
*D. iridis* lariat-capping ribozyme, MCM refined regions‡	69	17.0†	n.a.§	11.2	n.a.§

* MCM modeling was performed with MOHCA-seq constraints from datasets collected on the ligand-bound state; ligands were not included during Rosetta modeling.

† M^2^/Rosetta statistics are reported for RNA-puzzle submission rank 1 models, which included subdomains built by homology modeling.

‡ Calculated over core domain residues or refined regions after alignment using MAMMOTH (Ortiz et al., 2002); see ‘Materials and methods’.

§ p-value computed using analytical formula for secondary-structure-constrained 3D modeling in (Hajdin et al., 2010); it is not applicable to peripheral domains. Value above 0.9 are not well-determined and are presented as > 0.9.

The originally published table is also shown for reference:

Table 1 Original. Benchmark of multidimensional chemical mapping on RNAs with crystal structures

**Table:** 

RNA	Length	M^2^/Rosetta		MCM	
		RMSD to crystal (Å) (accuracy)	p-value§	RMSD to crystal (Å) (accuracy)	p-value§
*Tetrahymena* ribozyme P4–P6 domain	158	38.3	1.0	8.6	5.1 × 10^−12^
*V. cholerae* cyclic-di-GMP riboswitch aptamer, ligand-bound*	89	11.3	0.28	7.6	4.3 × 10^−3^
*F. nucleatum* double glycine riboswitch ligand-binding domain, ligand-bound*	159	30.5	1.0	7.9	2.1 × 10^−13^
*S. thermophilum* adenosylcobalamin riboswitch aptamer, ligand-bound*	168	17.1†	3.9 × 10^−3^	11.9	1.5 × 10^−8^
Class I ligase	127	26.3	1.0	14.5	5.6 × 10^−2^
Class I ligase, core domain‡	87	14.0	0.86	11.1	0.30
*D. iridis* lariat-capping ribozyme	188	9.6†	1.2 × 10^−15^	8.2	<1.0 × 10^−16^
*D. iridis* lariat-capping ribozyme, MCM refined regions‡	69	17.0†	n.a.§	11.2	n.a.§

* MCM modeling was performed with MOHCA-seq constraints from datasets collected on the ligand-bound state; ligands were not included during Rosetta modeling.

† M^2^/Rosetta statistics are reported for RNA-puzzle submission rank 1 models, which included subdomains built by homology modeling.

‡ Calculated over core domain residues or refined regions after alignment using MAMMOTH (Ortiz et al., 2002); see ‘Materials and methods’.

§ p-value computed using analytical formula for secondary-structure-constrained 3D modeling in (Hajdin et al., 2010); it is not applicable to peripheral domains.

